# Identification and functional study of a novel *FOXC1* missense mutation in a Chinese family with Axenfeld–Rieger syndrome

**DOI:** 10.1038/s41598-025-04872-x

**Published:** 2025-06-06

**Authors:** Xue Gong, Xinlan Lei, Zhaohui Li, Yiqiao Xing

**Affiliations:** 1 Eye Center, Renmin Hospital of Wuhan University, No. 99 Zhang Zhidong Road, Wuhan, 430060 Hubei China; 2 Aier Eye Hospital of Wuhan University, No. 481 Zhongshan Road, Wuhan, 430064 Hubei China

**Keywords:** *FOXC1*, Axenfeld–Rieger syndrome, Missense mutations, 3D protein structure, Molecular dynamics simulation

## Abstract

Axenfeld–Rieger syndrome (ARS) is an uncommon manifestation of anterior segment development dysregulation. We recruited a two-generation Chinese family with hereditary ARS, which manifested as posterior embryotoxon, corneal leucoplakia, ectopic pupil, and extensive iris stromal atrophy with iridocorneal adhesion. Whole-exome sequencing (WES) was utilized for the initial screening, followed by Sanger sequencing to identify pathogenic gene mutations. A novel missense variant in *FOXC1* (c.382C > T, p.H128Y) was identified. The FOXC1 protein is highly evolutionarily conserved. The potential effects of this mutation on protein structure were studied using 3D modelling and molecular dynamics (MD) simulation techniques. 3D modelling revealed that the mutation altered the conformation of the FOXC1 protein. The MD results suggested that the mutation could reduce the stability of the protein structure. Western blotting revealed that the expression of the mutant FOXC1 (c.382C > T, p.H128Y) was lower than that of the wild type. In summary, a novel *FOXC1* variant was discovered within a Chinese family with ARS, broadening the spectrum of ARS mutations. This study also elucidated the underlying mechanisms by which *FOXC1* gene deficiency causes ARS.

**Supplementary Information:**

The online version contains supplementary material available at 10.1038/s41598-025-04872-x.

## Introduction

Axenfeld–Rieger syndrome (ARS) is a relatively uncommon genetic disorder distinguished by abnormal development of the anterior ocular segments. ARS affects approximately 1 in 50,000–100,000 births^1^. The characteristics of ARS include posterior embryotoxon, corneal abnormalities, iris hypoplasia or aniridia, iris atrophy, iris anterior adhesion, eccentric pupils or pupil malformations, and pupillary pigment eversion^2^. Optic nerve abnormalities or macular retinoschisis occur in a few patients^3,4^. Additionally, patients with ARS may exhibit a range of extraocular clinical symptoms. The most prevalent morphological anomalies include craniomaxillofacial deformities, tooth malformation, and redundant umbilical skin^2^. In certain cases, hearing loss, mental retardation, cardiac anomalies, and cerebellar malformation may occur^5^. Moreover, approximately fifty percent of individuals diagnosed with ARS experience glaucoma in the latter stages because of anomalies in the structures for aqueous humour drainage^6,7^.

ARS can be categorized into three subtypes. Type 3 (OMIM 602,482) is characterized mainly by isolated ocular defects or hearing loss, as well as cardiac defects caused by heterozygous variants of forkhead box C1 (*FOXC1*)^5^. Forty to seventy percent of ARS cases are caused by pathogenic variants in the *FOXC1* or pituitary homeobox 2 (*PITX2*) genes^8,9^. FOXC1 and PITX2 are two transcription factors that physically interact with each other to form a high-order transcription factor complex rather than acting alone^10^. PITX2 represses FOXC1 and negatively regulates its transcription. This intricate regulatory mechanism may explain the phenotypic similarities observed in patients carrying two different gene mutations. Additionally, studies have revealed several chromosomal loci and genes involved in ARS onset and progression, including 13q14^11^, 16q24^12,13^, *CYP1B1*^14^, *PRDM5*^15^, *COL4A1*^16^, *ADAMTS17*^17^, and *PAX6*^18^.

The *FOXC1* (MIM 601,090) gene lies on chromosome 6p15.3 and is important for embryonic and eye development^19^. To date, over 100 pathogenic variants in *FOXC1* have been discovered in ARS patients^4^. Among these variants, missense mutations are the most common, especially in ARS patients with eye symptoms alone. Previous reports have shown that missense mutations are associated with ARS caused by the loss of function of FOXC1^20^. Further investigation is essential to assess how FOXC1 missense variants influence molecular structure and protein functionality.

In this study, we identified a previously unreported *FOXC1* missense variant (c.382C > T) within a Chinese ARS (type 3) family through whole-exome sequencing. Further research was conducted from two perspectives. First, 3D modelling and molecular dynamics (MD) simulation analyses were conducted to explore the intramolecular effects of the novel variant on protein structure. Then, expression vectors for wild-type and mutant-type FOXC1 were constructed and transfected into cell lines to compare protein expression levels. Our findings expand the range of *FOXC1* mutations in the Human Gene Mutation Database (HGMD). Additionally, the genetic characterization of the *FOXC1* mutation provides novel paths for future diagnosis and treatment as well as important insights into the pathophysiology of ARS.

## Materials and methods

### Ethics statement

Ethical approval for this study was obtained from the Research Ethics Committee of the Aier Eye Hospital of Wuhan University and adhered to the guidelines of the Declaration of Helsinki of 1964 (ethical approval number: 2023IRBKY120906, date of approval: December 9, 2023). Prior to the commencement of the study, all participants consented to participate and provided a signed informed consent document.

### Patients and clinical evaluations

An ARS family with three members (two affected, one unaffected) was recruited from the Aier Eye Hospital of Wuhan University. None of the family members had previously married close relatives. Furthermore, female participants had a typical pregnancy history, defined as the absence of complications during pregnancy or delivery, no use of teratogenic medications, and no known exposure to teratogenic substances. Each participant underwent a thorough eye examination, which included best-corrected visual acuity (BCVA) assessment, optometry, intraocular pressure determination, slit-lamp examination, anterior eye segment photography, gonioscopy, and fundoscopy. A general physician assessed each participant for the existence of systemic diseases.

### Whole-exome sequencing and sanger sequencing validation

Peripheral blood (2 ml) was collected from each family member into ethylenediaminetetraacetic acid (EDTA) tubes. Genomic DNA was extracted from the EDTA whole blood samples with a DNA extraction kit (Tiangen Biotech, Beijing, China) following the manufacturer’s protocols. A genomic library was constructed, and the relevant target gene exons and adjacent intron regions were captured through probe hybridization for enrichment. The enriched target gene fragments were sequenced on a NovaSeq 6000 (Illumina, California, USA) using the paired-end sequencing strategy. Raw data quality control included the following steps: reads with > 50% of bases having a quality value < 5, reads shorter than 150 bp, and reads containing > 15 N bases were filtered out. The average sequencing depth exceeded 100 x, with a Q30 base percentage > 85%, and ≥ 95% of regions achieved ≥ 20 × coverage. Processed reads were aligned to the human hg19 (GRCh37/hg19) reference genome with the Burrows–Wheeler Aligner^21^. Afterwards, the data were sorted with SAMtools and Picard. Single-nucleotide polymorphisms and insertion‒deletions were detected and annotated with GATK and ANNOVAR, respectively^22^. Variants with a frequency < 5% in the 1000 Genomes Project (http://www.1000genomes.org/) and an allele frequency < 0.1% in the gnomAD database (http://gnomad.broadinstitute.org/) were extracted. Protein functions caused by these genetic mutations were analysed using the protein harmfulness prediction programs SIFT (https://sift.bii.a-star.edu.sg), PolyPhen2 (http://genetics.bwh.harvard.edu/pph2/) and MutationTaster (http://www.mutationtaster.org/). The deleteriousness of variant sites was assessed using various databases, such as OMIM, HGMD, and ClinVar. The variants were classified according to the American College of Medical Genetics and Genomics (ACMG) guidelines^23^. Sanger sequencing was used for verification of confirmed pathogenic variants.

### Multiple-sequence alignment, molecular modelling, and molecular dynamics simulations

The amino acid sequences of FOXC1 homologues across different species were sourced from the National Center for Biotechnology Information (NCBI) (http://www.ncbi.nlm.nih.gov) and were aligned using ESPript 3.0 (http://espript.ibcp.fr/ESPript/ESPript/). The wild-type FOXC1 protein sequence was uploaded to AlphaFold (https://alphafold.ebi.ac.uk/) to predict the structure of the protein^24^, and PyMOL (The PyMOL Molecular Graphics System, Schrödinger, LLC., New York, NY, USA) was utilized to visualize single point mutations and hydrogen bond networks. Then, MD simulations were implemented for both models using the GROMACS 2019.6 package^25^ with the AMBER99SB protein force field^26^. The TIP3P water model was applied^27^. The temperature was controlled at 300 K using the V-rescale method, and the pressure was held constant at 1 atm via the Parrinello‒Rahman pressure coupling technique. The LINCS algorithm was utilized to constrain the hydrogen bonds, incorporating an integration timestep of 2 fs, and the particle mesh Ewald (PME) methodology was employed to calculate the electrostatic interactions. A cut-off distance of 10 Å was selected for nonbonded interactions, with the neighbour list being refreshed every 20 steps. Energy minimization was implemented through the steepest descent method to eliminate overly close contact between atoms. The temperature of all the atoms within the system was subsequently increased to 300 K within 100 ps. A MD simulation lasting 100 ns was subsequently performed on the system, and the conformation was captured every 20 ps. After the simulation, simulation trajectories were generated and calculated using the standard tools of the GROMACS package^28^.

### Plasmid construction, cell culture, and transfection

The cDNA sequences of the wild-type and mutant-type FOXC1 used in this study were provided by General Biol Co., Ltd. FLAG-tagged amplified cDNA fragments were inserted between the HindIII and BamHI restriction sites of the pcDNA3.1 vector, which carried an EGFP reporter. Human embryonic kidney (HEK) 293 T cells were procured from the American Type Culture Collection (ATCC) cell bank and cultured in Dulbecco’s modified Eagle medium (Gibco, Life Technologies, USA) supplemented with 10% foetal bovine serum (Gibco, Life Technologies, USA) and 100 mg/ml of both penicillin and streptomycin at 5% CO2 and 37 °C^29^. The cells were then transfected with wild-type, mutant, or empty vectors using Lipofectamine 2000 (Invitrogen, Life Technologies) as directed by the manufacturer.

### Immunofluorescence

HEK293T cells were seeded at a density of 1 × 10^5^ cells per well into 24-well plates pretreated with cell climbing slides and allowed to incubate for 24 h before transfection. The cells were subsequently transfected with the pcDNA3.1 plasmid and the wild-type and mutant plasmids. The transfection efficiency and cellular localization of the plasmid were evaluated after 24 h and 48 h. To determine the cellular localization of the FOXC1/EGFP fusion protein, the cells were treated with 4% paraformaldehyde for 15 min, permeabilized and blocked with 0.5% BAST at ambient temperature for 1 h. The cells were incubated overnight at 4 °C with the primary FLAG tag antibody (1:1000, GB15938, Servicebio, China). After washing three times with PBS, the cells were incubated with a Cy3-conjugated secondary antibody (1:500, GB21301, Servicebio, China) for 1 h at ambient temperature. The cells were incubated in DAPI for 3 min. Images were taken using fluorescence microscopy (Olympus, IX73).

### Western blotting

HEK293T cells were transfected with the pcDNA3.1 plasmid and both the wild-type and mutant plasmids. After 48 h of transfection, total protein was extracted from the transfected HEK293T cells using RIPA lysis buffer (G2002; Servicebio, China) supplemented with PMSF (G2008, Servicebio, China) and a cocktail of protease inhibitors (G2006; Servicebio, China). A BCA Assay Kit (G2026, Servicebio, China) was subsequently used to measure total protein concentrations. A total of 20 μg of protein was resolved with a 10% SDS‒PAGE gel, which was subsequently transferred onto polyvinylidene fluoride (PVDF) membranes. The membranes were blocked in 5% skim milk at ambient temperature for 1 h before being subjected to immunoblotting with a mouse anti-FLAG tag antibody (1:1000, GB15938, Servicebio, China) and a mouse anti-GAPDH antibody (1:4000, GB15004, Servicebio, China) overnight at 4 °C. Afterwards, the membranes were probed with an HRP-conjugated goat anti-mouse IgG (H + L) secondary antibody (1:5000, GB23301, Servicebio, China) for 1 h at ambient temperature. Protein expression was visualized with enhanced chemiluminescence (ECL) reagents (G2014, Servicebio, China) and an imaging system (GelView 6000Plus, Guangzhou Biolight Biotechnology Co., Ltd.). The signal data were processed using ImageJ software, and each western blotting experiment was repeated a minimum of three times.

## Results

### Clinical findings

**Fig. 1:**
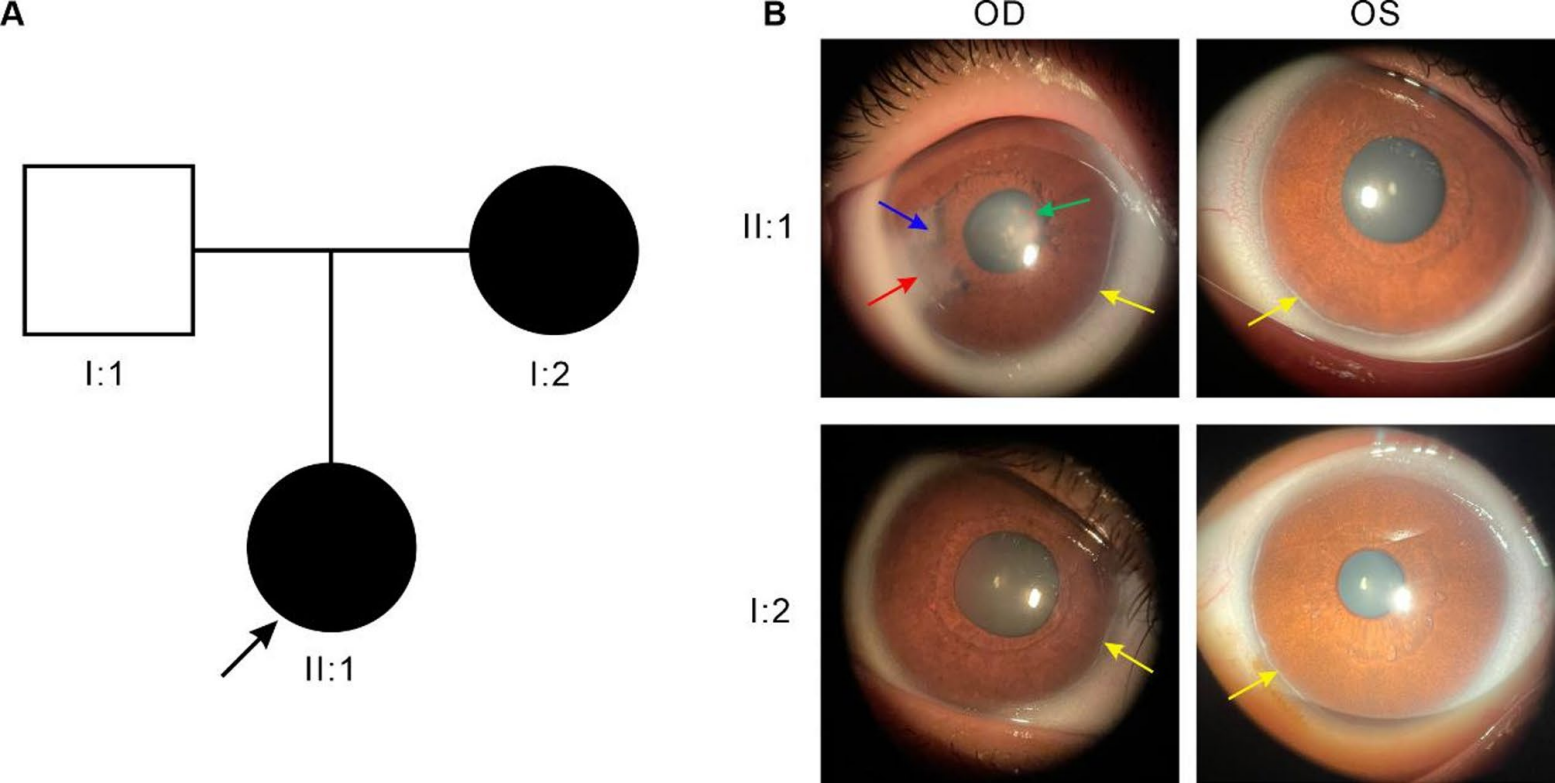
Pedigree of the family and clinical findings. (**A**) Two-generation pedigree of autosomal dominant congenital Axenfeld–Rieger syndrome. Squares denote male individuals, and circles represent female individuals. Filled symbols signify affected members, whereas empty symbols denote those who are unaffected. The proband is marked by a black arrow. (**B**) Yellow arrows indicate posterior embryotoxon, which can be observed 360 degrees from the limbus. The blue arrow indicates iridocorneal adhesions, iris atrophy, thinning and hole formation; the red arrow points to corneal leucoplakia; and the green arrow indicates adhesions between the iris and the lens capsule. OD stands for the right eye, and OS indicates the left eye.

The two-generation family tree included two affected members and one unaffected member, indicating autosomal dominant inheritance (Fig. 1A). The proband (II:1) was a nine-year-old Chinese girl who visited the Aier Eye Hospital of Wuhan University with complaints of poor vision. Slit lamp examination and anterior segment photography revealed posterior embryotoxon of anterior segment abnormalities in both eyes of the proband (II:1) and the proband’s mother (I:2). In addition, corneal leucoplakia, an ectopic pupil, and extensive iris stromal atrophy with iridocorneal adhesion were observed in the right eye of the proband (II:1) (Fig. 1B). Both the proband and her mother exhibited normal intraocular pressure, and physical examination indicated no abnormalities in the heart, abdomen, hearing or dental morphology. The proband’s father, I:1, exhibited no ocular or systemic symptoms.

### Identification of FOXC1 mutations

**Fig. 2:**
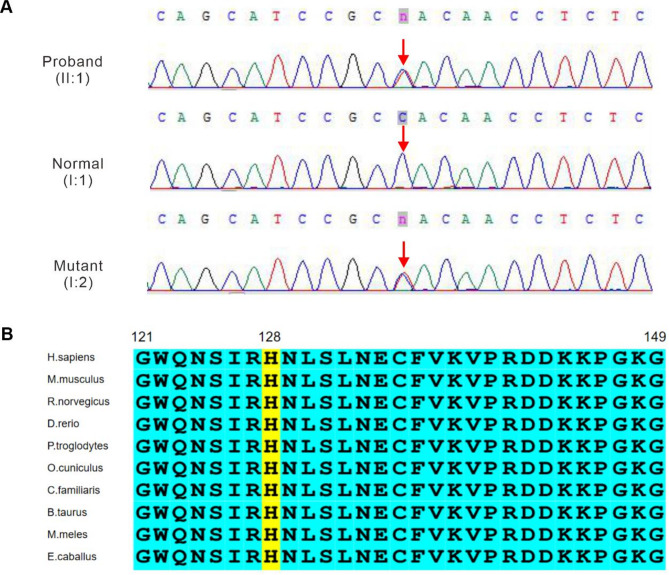
Sanger sequencing validation and multiple sequence alignment among different species. (**A**) Identification and confirmation of the *FOXC1* heterozygous missense mutation (c.382C > T) in the ARS family. The mutation in *FOXC1* is indicated by red downwards arrows. (**B**) Multispecies alignment showing significant conservation of the amino acid affected by the novel missense mutation.

To identify the causative mutation(s), we collected the proband’s DNA for WES, which revealed a novel missense variant (NM_001453.3:c.382C > T:p.His128Tyr) in *FOXC1* (Fig. 2A). We validated the *FOXC1* candidate mutation in the parents by Sanger sequencing of genomic DNA. The sequence data have been deposited in the Sequence Read Archive database repository with the accession number PRJNA1126424. This point mutation leads to a substitution of one amino acid, changing a histidine (H) to a tyrosine (Y) at position 128. According to the ACMG criteria, this variant has been classified as having uncertain significance. The findings of the three bioinformatics prediction programs (PolyPhen2, SIFT, and MutationTaster) were consistent with the deleterious effects of the mutation. An analysis of evolutionary conservation indicated that the amino acid located at the mutation site was highly conserved across multiple species (Fig. 2B).

### Protein 3D structure prediction and molecular dynamics simulation analyses

**Fig. 3:**
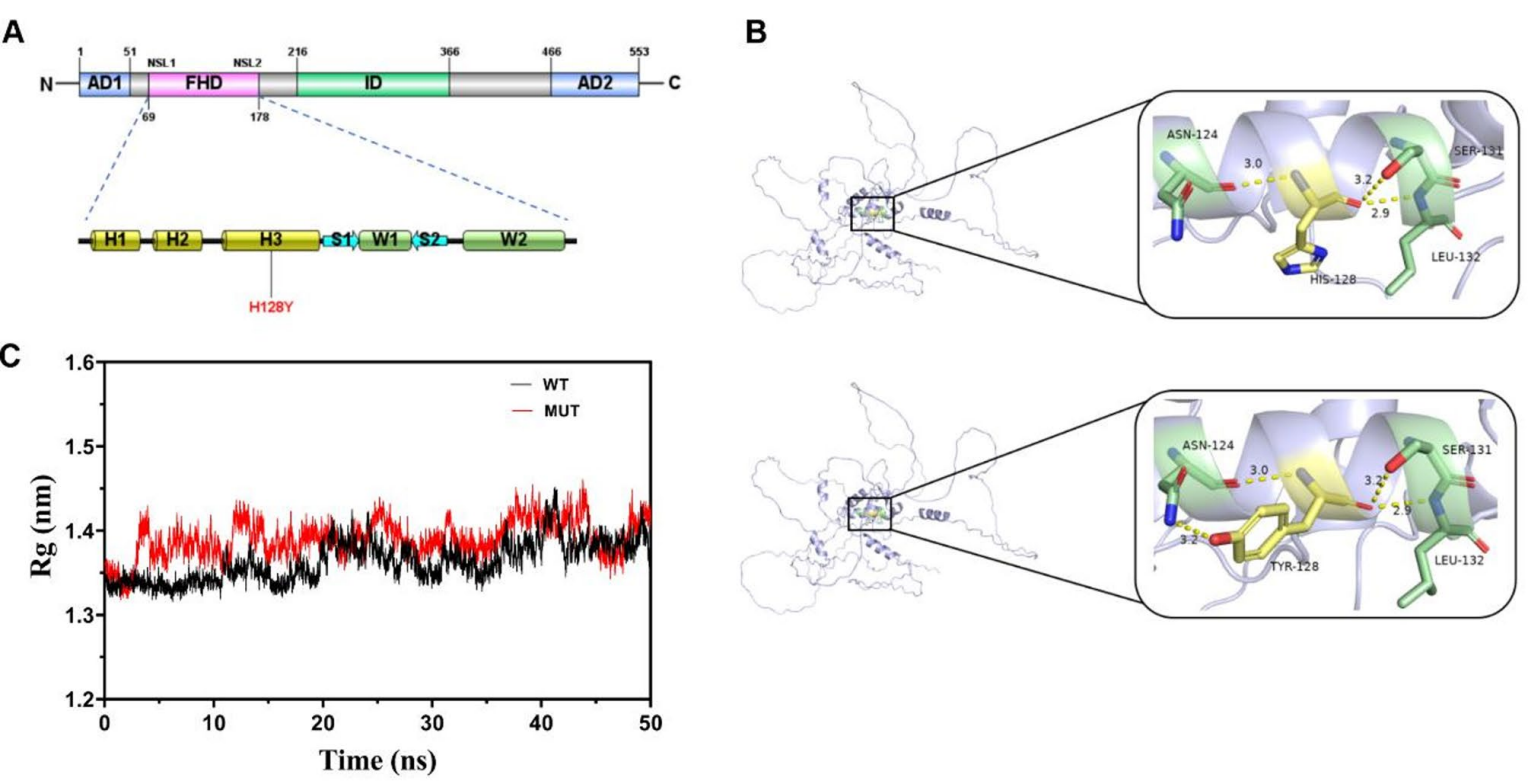
FOXC1 protein domains, 3D modelling structures of the FOXC1 wild-type (FOXC1-WT) and FOXC1 mutant-type (FOXC1-H128Y) proteins, and molecular dynamics (MD) simulation results. (**A**) Representation of the human FOXC1 protein and its domains. The mutation site is located within ɑ-helix 3 of the forkhead domain. (**B**) 3D modelling structures of domains containing FOXC1-WT and FOXC1-H128Y. The interacting residues are illustrated in stick format, and hydrogen bonds are represented by yellow dashed lines. (**C**) The radius of gyration (Rg) for the FOXC1-WT and FOXC1-H128Y proteins calculated from MD simulations. WT, FOXC1 wild-type; MUT, FOXC1-H128Y.

The mutation site H128Y is located on ɑ-helix 3 of the forkhead domain (FHD) of the FOXC1 protein (Fig. 3A); the mutation changes a positively charged histidine into an uncharged tyrosine. In the FOXC1 wild-type (FOXC1-WT) protein model, His128 establishes three hydrogen bonds with the binding residues Asn124 (distance, 3.0 Å), Ser131 (distance, 3.2 Å), and Leu132 (distance, 2.9 Å). In the FOXC1 mutant-type (FOXC1-H128Y) protein model, an additional hydrogen bond is formed between Tyr128 and Asn124 (distance, 3.2 Å) (Fig. 3B). The MD simulation results revealed that the radius of gyration (Rg) of FOXC1-H128Y was slightly greater than that of FOXC1-WT (Fig. 3C).

### *Cellular localization and expression of the FOXC1 mutant* protein

**Fig. 4:**
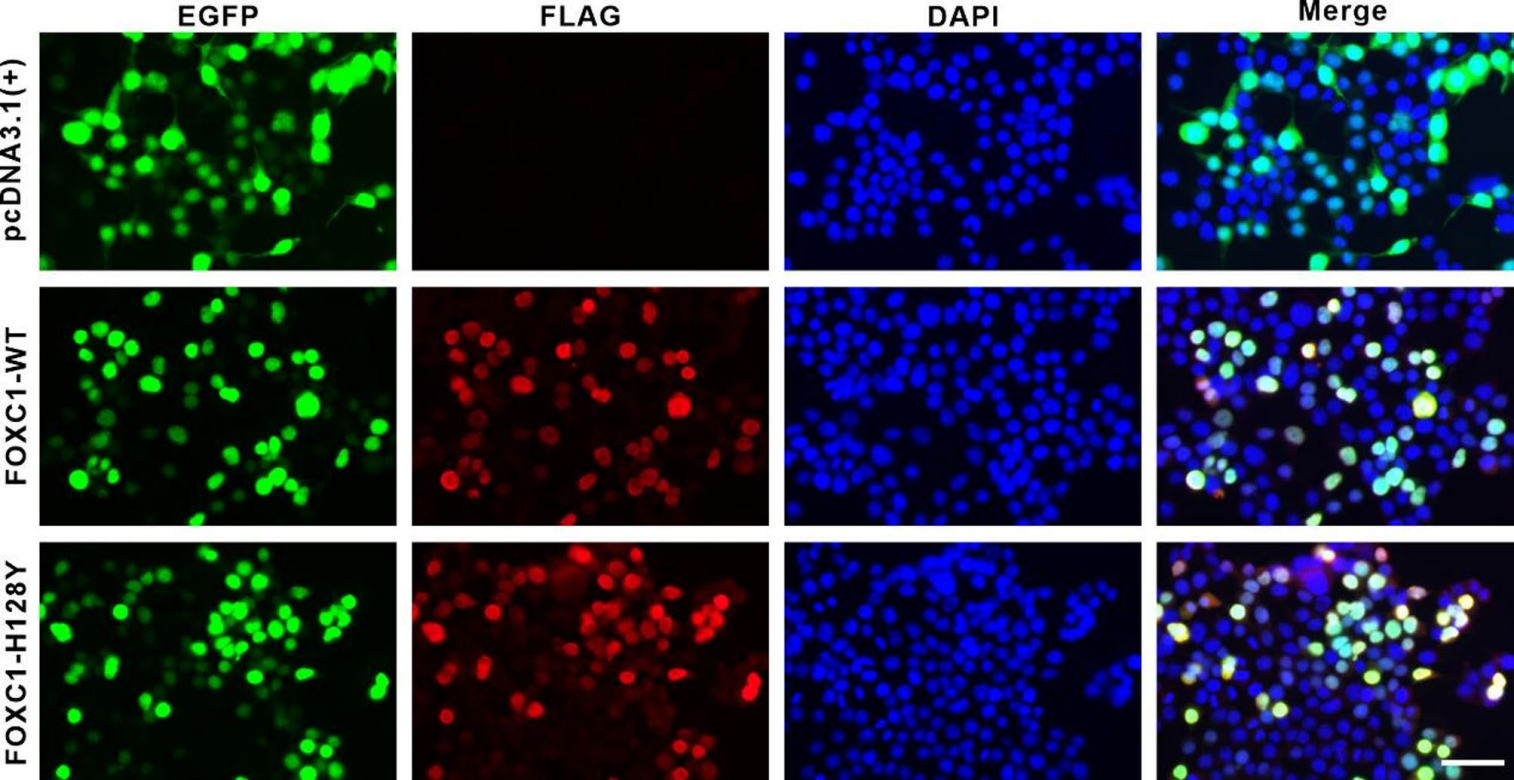
Images of the subcellular localization of the wild-type and mutated FOXC1 proteins. HEK293T cells were treated with a FLAG tag antibody and a Cy3-conjugated (red) secondary antibody; DAPI (blue) was used to counterstain the nucleus. No red fluorescence was detected in HEK293T cells without exogenous gene transfection. In cells that were transfected with the FOXC1-WT and FOXC1-H128Y plasmids, red fluorescence appeared predominantly in the nucleus. Scale bar = 100 µm.

**Fig. 5:**
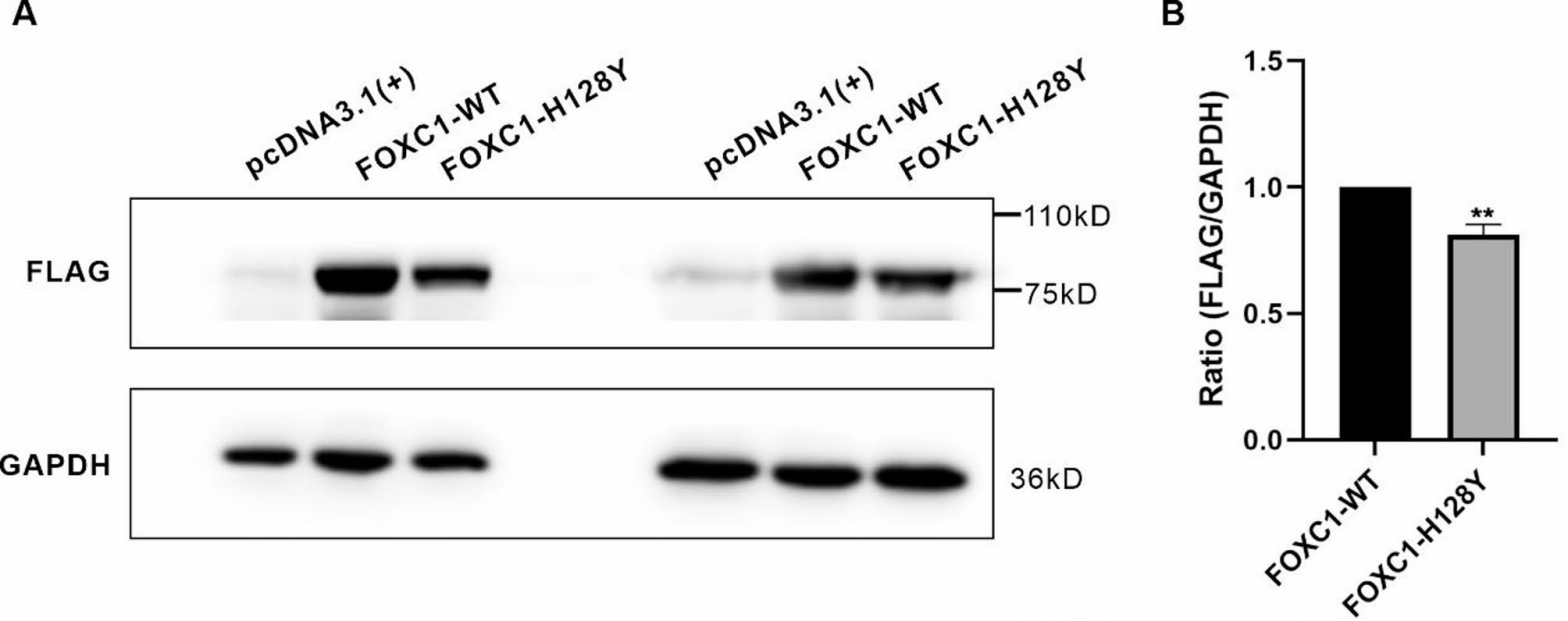
FOXC1-WT and FOXC1-H128Y expression. Western blot analysis revealed that the protein level of FOXC1-H128Y was lower than that of FOXC1-WT. (**A**) Total cell lysates from transfected HEK293T cells were subjected to western blot analysis. (**B**) Bar chart showing FOXC1-WT and FOXC1-H128Y protein expression levels (n = 4 independent experiments, *p* < 0.05 as determined by the Mann‒Whitney test, means ± SEs).

To evaluate whether the single-point mutation p.H128Y affects FOXC1 subcellular localization in vitro, we constructed FOXC1-WT and FOXC1-H128Y vectors and transfected them separately into HEK293T cells. The *FOXC1* gene was tagged with FLAG, which is a robust and commonly used epitope. Immunofluorescence staining revealed that the FOXC1-WT and FOXC1-H128Y proteins were efficiently expressed in HEK293T cells, and both proteins were localized in the nucleus, indicating that this mutation does not affect the nuclear localization of the protein (Fig. 4). To further assess the expression of the FOXC1 protein, we extracted total protein from the cells and conducted western blotting with a FLAG antibody. The western blotting results revealed a reduction in the FOXC1-H128Y protein level compared with that of the FOXC1-WT protein (Fig. 5).

## Discussion

In this study, a novel missense *FOXC1* variant (c.382C > T, p.H128Y) was discovered in a Chinese family affected by hereditary ARS using WES and verified by Sanger sequencing. *FOXC1* encodes a transcription factor crucial for eye development, and pathogenic variants of *FOXC1* have been linked to ARS. Several protein function prediction programs (PolyPhen2, MutationTaster, and SIFT) predicted that this missense variant has a negative impact. The His128 residue is highly evolutionarily conserved across diverse species, suggesting its importance for protein function. The results from 3D structural modelling indicate that the H128Y variant may lead to the establishment of extra hydrogen bonds, resulting in a change in the protein’s conformation. MD simulation results for the protein models suggested that the Rg of the FOXC1-H128Y model was slightly larger than that of the FOXC1-WT model. Rg has been shown to be a critical parameter associated with protein stability^30^. We propose that this mutation might increase the local volume of the protein, resulting in loose protein structures and reduced protein stability. Moreover, we constructed FOXC1-WT and FOXC1-H128Y plasmids and transfected them into HEK293T cells to examine the impact of the variant on cellular localization and protein expression in vitro. Both the FOXC1-H128Y and FOXC1-WT proteins localized to the nucleus, indicating similar subcellular positions. However, western blot analysis revealed decreased expression of FOXC1-H128Y compared with that of FOXC1-WT. Consequently, the c.382C > T mutation resulted in conformational changes and reduced protein expression of the FOXC1-H128Y variant, potentially contributing to the pathogenesis of ARS.

*FOXC1* belongs to the FOX family and encodes the FOXC1 protein, which functions as a critical regulatory factor in controlling embryonic and ocular development^31^. Cranial neural crest cells in the periocular mesenchyme form the cornea, iris, trabecular meshwork, sclera, orbital bones, and cartilage during embryogenesis^32,33^. Shields proposed that ARS occurs when neural crest cell development is halted during the latter stages of pregnancy, resulting in primitive endothelial cells remaining in the iris tissue and embedding in the anterior chamber angle^34^. In animal experiments, Smith^35^ reported that *Foxc1*^+*/-*^ mice presented multiple obvious abnormalities that were analogous to those of human ARS. These abnormalities were caused by mutations affecting the function of FOXC1 in governing the migration and survival of neural crest cells.

To date, dozens of *FOXC1* missense mutations have been found to be involved in ARS. Most missense variants are located within the typically highly conserved FHD of FOXC1. FOX family members share highly conserved FHD sequences, which consist of 110 amino acids and form a conventional winged helix fold comprising three ɑ-helices and two wing-like loops^36^. In previous studies, several missense mutation positions in the FHD were shown to influence FOXC1 protein structure and function. Saleem et al. reported that the I87M mutation is located within the first ɑ-helix of the FHD, which may lead to a reduction in FOXC1 protein expression and stability. The I126M mutation is found in ɑ-helix 3 of the FHD, and while this mutation does not affect the DNA-binding capacity, it does lead to a decrease in transactivation ability. The S131L mutation results in a reduction in the DNA-binding capacity of FOXC1^37^. Medina‒Trillo et al. reported that the I126S mutation resulted in decreased protein stability, compromised DNA binding, and altered subcellular localization^38^. The DNA-binding specificity of FOXC1 is attributed to DNA recognition by ɑ-helix 3, which also plays a crucial role in the interaction with the main DNA groove^39^. Berry et al. identified two pathogenic mutations in *FOXC2* (R121H and S125L) in patients suffering from lymphedema with distichiasis (LD). These mutation sites are located on ɑ-helix 3 of the FHD in FOXC2 and are expected to disrupt the protein’s capacity for DNA binding and transcriptional activation^40^. In ɑ-helix 3 of the FOXC1 FHD, histidine residue 128 contributes to the development of a hydrophobic core that stabilizes the spiral structure. Through molecular analysis, Seifi et al. investigated the functional consequences of the H128R mutation in FOXC1. Their results demonstrated that H128R exhibited protein levels comparable to those of FOXC1-WT. However, only 42% of the H128R proteins displayed proper nuclear localization. Utilizing luciferase reporter systems, the study also revealed that, compared with empty vector controls, FOXC1-WT induced a 14-fold increase in activation, whereas H128R resulted in only a 2.2-fold increase in activation. The authors concluded that H128R disrupts the structure of ɑ-helix 3, thus impacting DNA binding and leading to a diminished capacity to initiate transcription^41^. In our experimental observations, the H128Y conformation of ɑ-helix 3 was altered by the formation of additional hydrogen bonds. Additionally, H128Y resulted in less compact structures according to the MD results. Therefore, we infer that the substitution of tyrosine for histidine may impact the DNA recognition capability of ɑ-helix 3, subsequently hindering the protein’s ability to bind DNA and initiate transcription. The H128Y mutant protein can easily translocate to the nucleus because the two nuclear localization sites (NLS1 and NLS2) on both termini are not affected. Another potential contributing factor to pathogenicity identified in our study was the reduced expression of the mutant protein. Some studies have indicated that *FOXC1* is highly sensitive to dosage; increased or decreased levels of functional FOXC1 protein have been shown to lead to ARS^35,42^. These findings indicate that precise regulation of gene expression is essential for normal ocular development. Importantly, within the pedigree in this study, the clinical manifestations differed significantly even among patients carrying the same variant. Modifier genes, differences in gene expression patterns, or environmental factors could explain the phenotypic differences in disease severity observed in this study^43^.

This study has several limitations. Additional animal model studies are necessary to elucidate the underlying mechanism by which the mutation in the FOXC1 gene causes a loss of function, resulting in ARS. Further investigations are needed to ascertain the target genes of *FOXC1* and the underlying regulatory mechanisms involved.

## Conclusion

In conclusion, we identified a new *FOXC1* variant (p.H128Y) within a Chinese ARS family. The changes in protein conformation and decreased production caused by this mutation suggest that the pathogenic mechanisms of ARS are mediated through reduced levels of functional FOXC1 protein. While further functional validation is warranted, our findings expand the *FOXC1* mutation spectrum and provide preliminary evidence for applications of this mutation in genetic counselling. These results lay the groundwork for the future development of targeted therapeutic strategies.

## Electronic supplementary material

Below is the link to the electronic supplementary material.

Supplementary Material 1

## Data Availability

The details of the variant analysed during the current study are available in the Sequence Read Archive database repository under the accession number PRJNA1126424 (https://www.ncbi.nlm.nih.gov/nuccore/PP895327).
