# Supplementary material for: Identification and functional study of a novel FOXC1 missense mutation in a Chinese family with Axenfeld–Rieger syndrome
Source: Sci Rep. 2025 Jun 6;15:19957. doi: 10.1038/s41598-025-04872-x (PMC12144153; doi:10.1038/s41598-025-04872-x)
Supplement: Supplementary file 1 — Supplementary Material 1 [file 41598_2025_4872_MOESM1_ESM.pdf]

Supplementary information of the manuscript by Gong et al. entitled “Identification and functional study of a novel *FOXC1* missense mutation in a Chinese family with Axenfeld–Rieger syndrome”

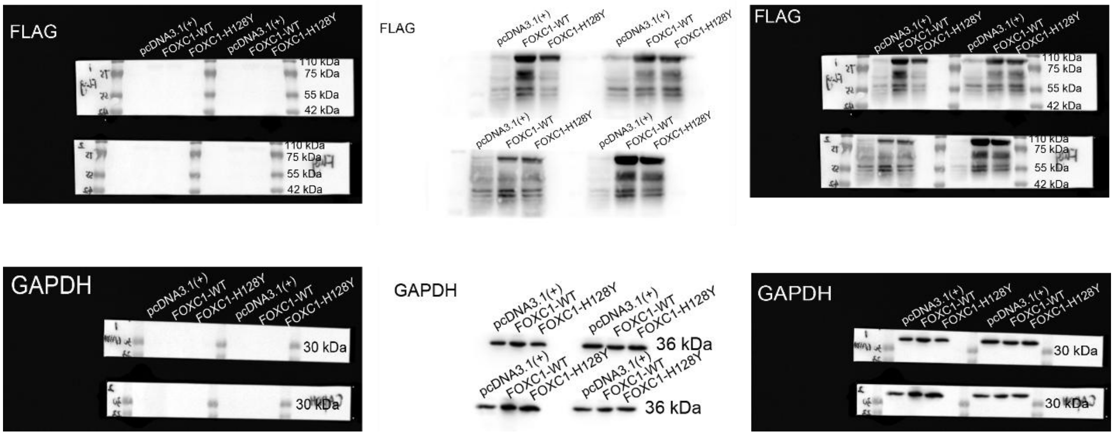

Supplementary Fig.1: Original images of the Western Blot (n=4 independent biological replicates).
